# Radiographic Evaluation of Bone Remodeling after Additively Manufactured Subperiosteal Jaw Implantation (AMSJI) in the Maxilla: A One-Year Follow-Up Study

**DOI:** 10.3390/jcm10163542

**Published:** 2021-08-12

**Authors:** Casper Van den Borre, Marco Rinaldi, Björn De Neef, Natalie A. J. Loomans, Erik Nout, Luc Van Doorne, Ignace Naert, Constantinus Politis, Hylke Schouten, Geert Klomp, Ludovic Beckers, Marshall M. Freilich, Maurice Y. Mommaerts

**Affiliations:** 1 Doctoral School of Life Sciences and Medicine, Vrije Universiteit Brussel, 1090 Brussels, Belgium; 2 Private Practice Villalba, 40122 Bologna, Italy; drmarcorinaldi@gmail.com; 3 Head of Department of Oro-Maxillo-Facial Surgery, General Hospital Oudenaarde, 9700 Oudenaarde, Belgium; Bjorn.DeNeef@azoudenaarde.be; 4 Private Clinic Face Ahead Antwerp, 2000 Antwerp, Belgium; loomansnatalie@me.com; 5 Division of Oro-Maxillo-Facial Surgery, GZA Hospitals, 2000 Antwerp, Belgium; 6 Oral and Maxillofacial Surgery, ETZ Hospitals, 5022 GC Tilburg, The Netherlands; e.nout@etz.nl (E.N.); klompgeert@gmail.com (G.K.); 7 Oral and Maxillofacial Surgery Cosmipolis Clinic Brugge, Ghent University Hospital, AZZENO, 8300 Knokke-Blankenberge, Belgium; vandoorne.luc@telenet.be; 8 Former Head Department of Prosthetic Dentistry, KU Leuven, Kapucijnenvoer 7, 3000 Leuven, Belgium; Ignace.Naert@uzleuven.be; 9 OMFS-IMPATH Research Group, Department of Oral and Maxillofacial Surgery, University Hospitals Leuven, 3000 Leuven, Belgium; constantinus.politis@uzleuven.be; 10 Department of Oral and Maxillo-Facial Surgery, Rode Kruis Ziekenhuis Beverwijk, 1566 NC Beverwijk, The Netherlands; mkachirurgie@gmail.com; 11 Academical Adviser of Elysee Dental, 3020 Herent, Belgium; ludovic.beckers@elysee-dental.be; 12 Division of Oral and Maxillofacial Surgery, Humber River Hospital, Toronto, ON M3M 0B2, Canada; drfreilich@rogers.com; 13 Private Clinic Orthoface Ghent, 9830 Ghent, Belgium; mauricemommaerts@me.com; 14 European Face Centre, Universitair Ziekenhuis Brussel, Vrije Universiteit Brussel, 1090 Brussels, Belgium

**Keywords:** maxilla, implantation, subperiosteal, alveolar bone loss, printing, three-dimensional, Cawood–Howell

## Abstract

Additively manufactured subperiosteal jaw implants (AMSJI) are patient-specific, 3D-printed, titanium implants that provide an alternative solution for patients with severe maxillary bone atrophy. The aim of this study was to evaluate the bony remodeling of the maxillary crest and supporting bone using AMSJI. Fifteen patients with a Cawood–Howell Class V or greater degree of maxillary atrophy were evaluated using (cone beam) computed tomography scans at set intervals: one month (T1) and twelve months (T2) after definitive masticatory loading of bilateral AMSJI implants in the maxilla. The postoperative images were segmented and superimposed on the preoperative images. Fixed evaluation points were determined in advance, and surface comparison was carried out to calculate and visualize the effects of AMSJI^TM^ on the surrounding bone. A total mean negative bone remodeling of 0.26 mm (SD 0.65 mm) was seen over six reference points on the crest. Minor bone loss (mean 0.088 mm resorption, SD 0.29 mm) was seen at the supporting bone at the wings and basal frame. We conclude that reconstruction of the severely atrophic maxilla with the AMSJI results in minimal effect on supporting bone. Reduced stress shielding with a biomechanically tuned subperiosteal implant does not induce radiographically significant crestal bone atrophy.

## 1. Introduction

Patients suffering from full edentulism have limited options for oral rehabilitation. Subperiosteal implants were introduced 80 years ago out of the need to improve stability and retention of full dentures in patients suffering from excessive ridge resorption [1]. At that time, cast frameworks and analogue radiographical imaging often led to inaccurate designs. The concepts of oral biology and stress shielding were not well understood at that time. These factors contributed to patient discomfort and a rather high complication rate [2]. The clinical use of subperiosteal implants was largely abandoned over time with the advent of screw-type endosseous titanium implants.

While endosseous implants are known to achieve osseointegration with a high degree of predictability, their placement and longevity are dependent on sufficient bone quality and quantity. Availability of an ideal osseous support may be compromised by excessive resorption or loss of the alveolar processes secondary to disuse atrophy, trauma, or neoplasia, which can render installation of endosseous implants risky or sometimes impossible [3,4,5]. Progression of alveolar bone resorption may continue following placement of screw-type implants, both in instances of placement into native alveolar bone and placement into regenerated bone following grafting procedures [5,6].

With the rise of digital technology, fabrication of patient-specific implants (PSI) has become possible. Disruptive 3D printing technology has led to the revisitation of earlier concepts such as subperiosteal implants.

**Figure 1:**
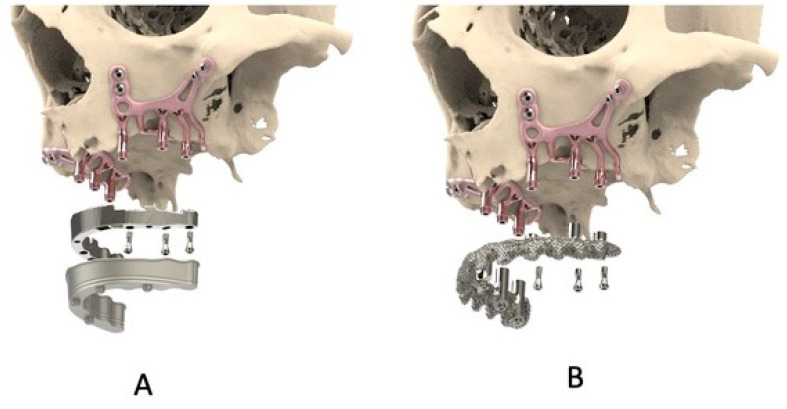
Visualization of the left and right AMSJI system and the connecting suprastructure. (**A**) The suprastructure is a 3D-printed titanium scaffold of a hybrid bridge. (**B**) The suprastructure consists of a double structure of an overdenture.

This has resulted in the innovation of the additively manufactured subperiosteal jaw implant (AMSJI) concept (CADskills BV, Ghent, Belgium). The AMSJI consists of two subunits (left and right). These are customized to the skeletal anatomy of each patient, based on a supplied CBCT data set. The subunits consist of two wings and a basal looped frame connecting the arms and the transmucosal posts. The wings are situated on the canine and zygomatic buttresses and are fixed in these locations using osteosynthesis screws (Figure 1). Both subunits are connected intra-orally by a temporary connector, and latter by a definitive primary matrix structure.

The AMSJI protocol constitutes a contemporary solution for a select group of patients with inadequate bone stock for screw-type endosseous implants (Figure 1) [7]. This tailor-made concept not only allows fixation onto the bone, but it also offers the patient an immediate functional restoration in a single intervention [8]. Clinical follow-up studies are necessary to confirm the efficacy of this contemporary concept.

The aim of this study was to evaluate the effect of reconstruction of the severely atrophic maxilla with the AMSJI on maxillary bone morphology in 15 patients.

## 2. Materials and Methods

A multicenter prospective study was designed by the International Workgroup on AMSJI. Patients were included in the study who were deemed to have Cawood–Howell maxillary alveolar atrophy of grade V or higher and wished to have maxillary rehabilitation with a fixed prosthesis. All surgeons received training using model surgery before operating their first AMSJI patient or by having their first case assisted by an experienced AMSJI surgeon. Patients were excluded from the study if they or the treating surgeon decided not to undergo a 1-year post loading CT scan, or if the CBCT was not a large enough dataset to allow full visualization of the AMSJI.

Perioperative antibiotic coverage consisted of amoxicillin–clavulanic acid, generally continued for five to seven days postoperatively.

Evaluation of the AMSJI position and effect on the surrounding bone was accomplished using CBCT imaging at one (T1) and twelve months (T2) after functional rehabilitation with the AMSJI. To ensure a consistently high quality of radiographic imaging, the following parameters were used: (1) conventional matrix: 512 rows × 512 pixels, (2) 90–120 kVp; (3) slice thickness was maintained between 0.5 and 0.7 mm, for CT, and 0.150 mm slice thickness for CBCT, and the same slice spacing was maintained throughout the scanning procedure; (4) each slice had the same display field, the same center of reconstruction, the same direction, and the same table height; (5) feed per rotation: max 1.0 mm; (6) reconstructed slice increment: maximum 1.0 mm; (7) the reconstruction algorithm for the bone was set at high resolution; and (8) Gantry tilt: 0°. All of the images were anonymized. Patients who were included as subjects in the study were assigned a code.

Postoperative CBCT images were stored as DICOM datasets. Data were imported into Materialise Medical 22.0 (Materialise, Leuven, Belgium) for segmentation. A threshold was chosen by the first author based on the suggested predefined threshold sets for “bone”. A 3D model was then generated. Semi-automated segmentation of the 3D model was established using a “region grow mode”, which was a feature of the analytical software. Manual 2D multi-slice segmentation was additionally performed to ensure meticulous removal of all of the titanium allow and scatter. The final files were saved in stereolithographic (STL) format using a “calculated part”. An “optimal or high quality” was selected.

The STL files were imported into Geomagic Studio 2018 (Geomagic, Morrisville, USA). Surface-based super-impositioning of the postoperative CT scans was carried out. Image fusion between the T1 and the T2 scan was obtained using a semiautomated registration process. Initial approximation was done by a manual overlap of the two images to achieve the best possible fit. Using the registration tool, images were aligned using the best-fit surface automatic alignment. With this method the software calculated the least possible distance between the two scans being compared, resulting in an automatic overlap of the scans to achieve the best fit based on the closest points.

**Figure 2:**
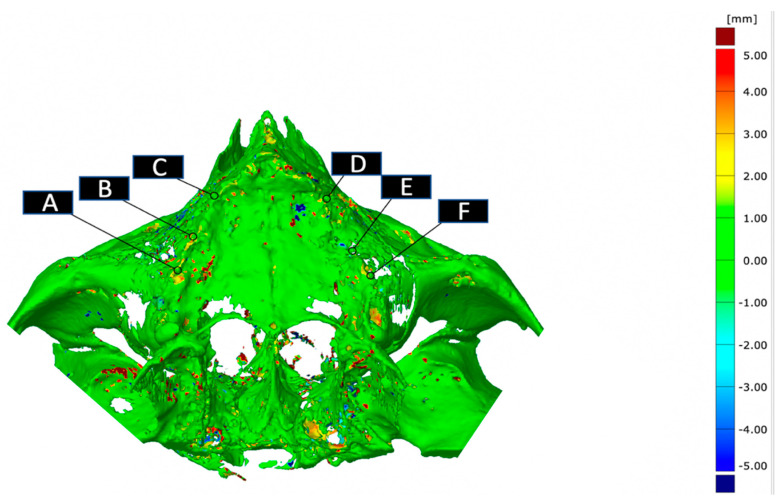
Bony surface reference points in the axis of the post. A–F indicates the reference points under the posts.

**Figure 3:**
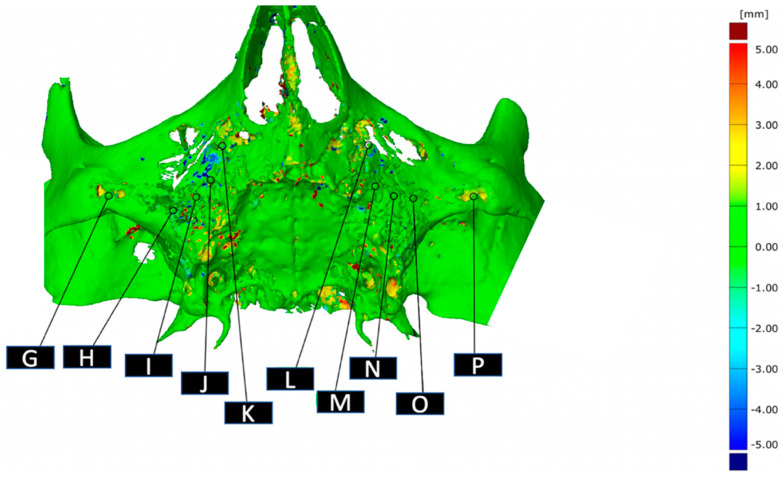
G, K, L, P: reference points under the wings; H, I, J, M, N, O: reference points under the basal looped frame.

Data were then again stored in STL format and imported in Gom Inspect Suite (Zeiss, Oberkochen, Germany). A color-code model was automatically generated for each patient to visualize the differences over time of bone apposition and resorption. Selected points on the color-code mask show the discrepancy (mm) between the fused images. On the crest, six bony reference points (A–F) were chosen in the axis of the posts (Figure 2). Four more reference points (G, K, L, P) were chosen between the two screws of the AMSJI wings on both sides (Figure 3). These points were determined based on, respectively, the perpendicular location of the posts projected on the crest and by determining the middle between the two screw holes. To assess the bone quantity perpendicularly underneath the basal looped frame, six more reference points were chosen between the connection of the neighboring posts and the looped frame (H–J; M–O, Figure 3).

Inter-rater reliability (parameters: single rater, consistency-agreement, two-way mixed-effects) and intra-rater reliability (two-way mixed-effects model) were calculated. The data were analyzed using SPSS version 27 (SPSS Inc., Chicago, IL, USA).

Ethical committee approval was obtained on 5 May 2019 (B.U.N 143201939806), and the principles stated in the Declaration of Helsinki and its later amendments were adapted. Informed consent was obtained from all individual participants.

## 3. Results

Nine males and six females with a mean age of 63.70 (SD 4.85) years were enrolled in this study. None of the patients complained of any discomfort, and none of the clinicians involved reported any complications at T1–T2. Surgeons and dentists were acquainted with implantology and charted results for both subjective and objective parameters. Objective and subjective parameters for success are the subject of another prospective study of the same International Study Group.

The status of the opposing mandibular dentition varied amongst subjects: two patients had a natural full arch dentition, and two had an interrupted arch without oral rehabilitation. Two additional patients had an interrupted natural arch with singular implants to replace lost molars. A fixed full arch prosthetic rehabilitation was observed in five patients. One patient had a fixed prosthesis supported by two mandibular AMSJIs and two singular implants at the canine regions. Three patients had removable frame prostheses.

No infections were reported at the time of recording. A total mean bone resorption of 0.26 mm (SD 0.65 mm) was seen over six reference points on the crest. Almost no bone loss (mean 0.088 mm resorption, SD 0.29 mm) was seen at the supporting bone at the wings and basal frame. Neutro-occlusion was established in each case. Table 1 and Table 2 report resorption and apposition of bone at the reference points.

Intraclass correlation coefficient (test–retest; two-way mixed effects model, type “consistency”) was 0.86, indicative of good reliability. Interclass correlation coefficient (two-way random, 95% interval, type ”consistency”) had an average value of 0.91 with a 95% confidence interval equal to 0.73–0.97, also showing good reliability between the assessors [9].

## 4. Discussion

Long-term survival of conventional subperiosteal implants has been documented [10,11,12]. Several reviews have, however, also reported on complications such as infections, early and late implant exposure, bone resorption, fistulation, and implant mobility, leading to considerable patient discomfort and implant failure [10,11,12,13,14,15]. The endosseous implants overcame several problems that had been experienced with the initial forms of subperiosteal implants and showed superior long-term results with minor patient discomfort [13,14,15,16]. Due to the feasibility of manufacturing in large quantities and the ease of installation and removal in the event of failure, endosseous implants became the first treatment of choice in the 1980s.

Despite their benefits, however, endosseous implants cannot be relied on by the surgeon to treat every clinical scenario. Adequate bone volume and quality are necessary to correctly position endosseous implants [17]. If sufficient bone volume and quality are not present, the endosseous implant is prone to fail, may damage important structures, or may be impossible to install [18]. The use of narrow and short implants represents an alternative option, but if significant bone volume is lost in height and width, even these cannot be used [19].

Various regenerative techniques may be performed to augment the alveolar ridge in both the vertical and horizontal dimensions if insufficient bone volume precludes implant placement; however, this option is limited to cases where sufficient native bone exists to support the grafts. Autologous bone harvesting can be accomplished from a variety of anatomic sites, allowing for placement of onlay grafts. This approach is often used and is considered by some authors as the gold standard of regeneration techniques [20,21,22].

One of the main drawbacks to the utilization of free grafts is that during harvesting microcirculation is unavoidably severed, impeding reestablishment of graft circulation. Revascularization of the graft must occur to ensure osteogenesis and graft survival. This process requires time, during which osteocyte vitality is frequently compromised [23]. Consequently, small areas of dead bone will form, leading to undesirable and unpredictable graft resorption. The amount of resorption is strongly dependent on the site from which the bone was harvested. According to the literature, mandibular block grafts utilized for maxillary ridge augmentation have resorption rates between 5 and 28% [22,24,25]. Onlay grafts harvested from the iliac crest have been shown to exhibit 50% average volume decrease 6 months following placement in the atrophied maxilla [26]. Fourcade et al. (2019) investigated the resorption of calvarial (parietal) and ramic bone grafts for pre-implant reconstruction of maxillary alveolar ridges and found a mean resorption of 25% for both types of block grafts [27].

Guided bone regeneration (GBR) is frequently performed with bone graft procedures to mitigate these high resorption rates. With this approach, bone substitutes and membranes are used in addition to harvested autologous grafts to exclude non-osteogenic cell populations. As a result, osteoblast cell proliferation is promoted, and connective tissue and epithelial cells are mechanically excluded, resulting in lower resorption rates and higher bone volume following ridge augmentation [28,29].

Despite the reported variable rates of bone graft resorption, the success ratios following implant placement in grafted bone are high. Aghaloo et al. (2016) performed a literature review evaluating implant outcomes following bone grafting of completely edentulous maxillae [22]; 2446 implants were placed, with follow-up ranging from 1 to 12 years. The range of implant survival rate in this review was 73.3–100%. When GBR was performed, the reported survival rate improved to 96.1–100%. Motamedian et al. (2016) found a success rate between 72.8% and 100% of 2.647 implants when onlay grafting was performed using autologous blocks in an atrophied maxilla [30].

In addition to onlay grafting and guided bone regeneration, maxillary sinus floor elevation remains a popular method for augmenting bone volume; however, this is a technique-sensitive procedure. A frequent reason for failure is intraoperative rupture of the Schneiderian membrane. This is a common occurrence, with a reported incidence of perforation ranging from 3.6% to 41.8% [31]. If lacerated, the membrane cannot perform the function of graft containment, which impedes osteogenesis [32].

Augmentation of the maxillary sinus floor limits the anterior extent of maxillary reconstruction to the premolar level. Atrophied maxillae often present with severe ridge atrophy at the premaxillary region as well, and this aspect of the deficient ridge is often not amenable to augmentation. Despite this limitation, several studies have claimed high implant success rates (>90%) with follow-up periods from 1 to 11 years [22,33].

Bone regenerative techniques give excellent results in the context of long-term implant success in the atrophied maxilla; however, there remain several disadvantages to this approach. Bone augmentation relies on osteogenic potential, which varies among patients. This potential diminishes with age, which could lead to a higher degree of resorption [34]. If substantial resorption occurs, esthetic and functional stability of the implants can be influenced. This may necessitate repetition of bone augmentation to ensure proper volume for re-implantation. There is also a relevant morbidity associated with the harvest and installation of block grafts at both the donor and recipient sites. The risk of dehiscence, infection, pain, swelling, neurosensory deficits, and graft failure remain pertinent [35]. In addition, the harvested blocks remain of finite thickness, rendering complete augmentation of a severely atrophied maxilla difficult. A staged approach may be necessary to increase the potential for implant survival.

Aghaloo et al. (2016) showed that simultaneous graft-implant placement results in an implant survival ranging from 73.7 to 91% compared with 88.9–100% when a staged approach was used [22]. Staging of procedures carries the requirement for, and disadvantages of, multiple surgeries, with twice the risk for post-surgical complications to arise. Following bone augmentation, patients need to be encouraged to limit usage of dentures due to increased risk of wound dehiscence, graft displacement, and graft resorption if early loading is permitted prior to graft incorporation. This encumbers the patient to remain edentulous between surgical treatments for up to 4 months [22].

The advent of titanium 3D printing and 3D planning software made reconsideration of the subperiosteal implant concept possible. CBCT imaging depicts a patient’s residual bone volume with considerable accuracy. CBCT data are used to generate a virtual bone model using reconstruction software. This facilitates the design of a subperiosteal implant with a high degree of accuracy for each patient.

Titanium (Ti) and its alloys are known to have excellent biocompatibility and are therefore widely used in medical and dental devices. This is in contrast with Vitallium (a cobalt–chromium–molybdenum alloy) used in the earlier lost-wax-technique subperiosteal implants, which has no soft tissue or bone integration properties. Bone resorption underneath rigidly fixed titanium alloy osteosynthesis plates is barely seen in the craniomaxillofacial skeleton [36]. Over 25% of osteosynthesis material used in both trauma and orthognathic surgery is overgrown by bone over time [36,37]. Such a phenomenon could contribute to an increase in stability and osseointegration of the AMSJI at the wings and basal frame. In our patients, sites of bony overgrowth were observed, predominantly at the upper parts of the wings. Beam hardening artefacts did not allow quantification.

Physiological alveolar ridge resorption in completely edentulous patients has been well documented [38,39,40]. The rate of resorption varies between patients, and strong variance of resorption has been shown at different times and sites within individual patients [41].

Initially, rapid bone loss occurs three months after tooth loss. This is followed by a slow but continuous resorption throughout life [40]. A systematic review and meta-analysis performed by Koodaryan and Hafezeqoran (2016) reported an average early bone loss associated with maxillary and mandibular implants of around 1.5 mm during the first year after the final restoration was installed [42]. A mean annual bone loss of 0.2 mm thereafter was seen. We anticipate that a similar trend of bone loss can be expected with AMSJI beyond the first year of function. Patients who received AMSJI-supported prosthetic rehabilitation showed a mean resorption at the alveolar ridge of 0.33 mm (SD 0.76 mm) and 0.08 (SD 0.33) mm at the wings and basal frame on the underlying zygo-maxillary bone one year post loading.

The amount of resorption is dependent on several variables. One of the factors that can contribute to this loss of bone is the type of oral rehabilitation [19]. Kovacić et al. (2010) measured the bone resorption at the maxillary alveolar ridge using radiographic measurements on lateral cephalograms in 31 completely edentulous individuals after five years of wearing complete dentures [41]. A mean bone loss of 0.79 mm was found over a period of 5 years. Atwood et al. (1971) found the mean alveolar ridge resorption to be around 0.010 mm per year; however, the general rate of resorption varied greatly between different individuals from 0 mm to 0.70 mm [43]. This phenomenon can account for some of the crest resorption seen in our series.

Another factor that could account for some resorption is the raising of the mucoperiosteal flap [44,45,46]. When alveolar bone becomes exposed, the underlying bone is partially deprived of oxygen, and osteoclastic activity is thereby promoted, resulting in bone resorption and subsequent remodeling [47,48]. For implantation of AMSJI, preparation of a mucoperiosteal flap is necessary to correctly fixate the system.

Maier FM (2018) studied the effect on crestal bone in the maxilla after implant placement using conventional mucoperiosteal flap elevation versus a flapless procedure [49]. After one year, a mean crestal bone loss of 0.55 ± 0.57 mm was seen in the conventional mucoperiosteal flap group (100 patients vs. 95 in the control group). Merheb et al. (2014) came to almost the same conclusions and found a mean resorption of 0.40 mm after full thickness flap elevation [48].

To accurately perform a fusion of the T1 and T2 images, and to visualize the effect of AMSJI on the underlying bone, a segmentation of the AMSJI from the underlying bone on the CBCT images was necessary prior to the fusion.

Several software packages are available, which in turn use different tools for segmentation; however, the principle remains the same. The stored DICOM file is imported into the chosen program, and the desired anatomical structures are 3D-rendered towards a 3D model. A threshold is manually chosen initially to segment out any unvoluntary voxels. Thresholding defines a range of grey values. If voxels fall into this range, they are included in the segmented object. This is done manually. After determining the threshold and visualizing the underlying skeleton, further segmentation is done manually to filter out scatter. If segmentation is inaccurate, an error is already incorporated into the surface and volume before effective analysis can commence. Machine learning could improve the segmentation process, and further automation could lower the risk of operator error hereby improving segmentation and analysis results [50].

The AMSJI remained a challenge for segmentation of the CBCT data due to several factors: (1) some patients showed low bone quality, which made it difficult to visualize the bone as a 3D model; (2) the Ti-alloy composition of the AMSJI causes beam hardening artefacts; (3) the presence of bordering soft tissue; (4) low contrast resolution of several of the CBCT datasets; and (5) reduced quantity of the underlying bone present in some cases. This method of semiautomated segmentation, including the factors listed above, rendered the measuring process potentially prone to operator error. In addition, a surface-based superimposition uses only the surface of the 3D structure for the overlapping. A high-quality surface is required for an accurate superimposition [51].

## 5. Conclusions

In this study, 15 patients were radiographically examined 1 and 12 months after masticatory rehabilitation based on bilateral AMSJI implantation in the maxilla. The effect on the supporting bone was evaluated. Minor atrophy was seen at the alveolar ridge, but minimal atrophy was detected under the fixation wings.

## Figures and Tables

**Table 1:** Effect on the bony alveolar ridge perpendicular underneath the posts (in mm). A negative value indicates a mean resorption.

AMSJI	Point	∆ Each Point T1–T2 (SD)	∆ Side T1–T2 (SD)	∆ AMSJI Total T1–T2 (SD)
	A	−0.29 (1.04)	−0.24 (0.85)	−0.26 (0.65)
Right	B	−0.24 (0.94)		
	C	−0.18 (0.80)		
	D	−0.33 (0.89)	−0.27 (0.53)	
Left	E	−0.46 (0.57)		
	F	−0.050 (0.55)		

**Table 2:** Effect of the AMSJI wings and basal frame on the underlying zygomaxillary bone (in mm). A negative value indicates a mean resorption, and a positive value indicates a mean apposition.

AMSJI	Point	∆ Each Point T1–T2 (SD)	∆ Side T1–T2 (SD)	∆ AMSJI Total T1–T2 (SD)
	G	0.32 (0.67)	−0.060 (0.40)	−0.088 (0.29)
	H	−0.040 (1.46)		
Right	I	−0.38 (0.71)		
	J	−0.18 (0.57)		
	K	−0.03 (0.57)		
	L	−0.18 (0.41)	−0.11 (0.26)	
	M	−0.44 (0.47)		
Left	N	0.010 (0.59)		
	O	0.060 (0.75)		
	P	−0.030 (0.58)		

## Data Availability

The data presented in this study are available on request from the corresponding author.

## References

[1] Dahl C. (1943). If the opportunity for implantation in the jaw of metal skeletons as the base or retention for fixed or removable dentures. Odontol. Tidskr..

[2] Linkow L.I., Ghalili R. (1998). Critical design errors in maxillary subperiosteal implants. J. Oral Implant..

[3] Fretwurst T., Nack C., Al-Ghrairi M., Raguse J., Stricker A., Schmelzeisen R., Nelson K., Nahles S. (2015). Long-term retrospective evaluation of the peri-implant bone level in onlay grafted patients with iliac bone from the anterior superior iliac crest. J. Cranio-Maxillofac. Surg..

[4] Duttenhoefer F., Nack C., Doll C., Raguse J.-D., Hell B., Stricker A., Nelson K., Nahles S. (2015). Long-term peri-implant bone level changes of non-vascularized fibula bone grafted edentulous patients. J. Cranio-Maxillofac. Surg..

[5] Rosén A., Gynther G. (2007). Implant Treatment without Bone Grafting in Edentulous Severely Resorbed Maxillas: A Long-Term Follow-Up Study. J. Oral Maxillofac. Surg..

[6] Kim Y.-K., Kim S.-G., Kim B.-S., Jeong K.-I. (2014). Resorption of bone graft after maxillary sinus grafting and simultaneous implant placement. J. Korean Assoc. Oral Maxillofac. Surg..

[7] Mommaerts M. (2017). Additively manufactured sub-periosteal jaw implants. Int. J. Oral Maxillofac. Surg..

[8] Mommaerts M. (2018). Evolutionary steps in the design and biofunctionalization of the additively manufactured sub-periosteal jaw implant ‘AMSJI’ for the maxilla. Int. J. Oral Maxillofac. Surg..

[9] Koo T.K., Li M.Y. (2016). A Guideline of Selecting and Reporting Intraclass Correlation Coefficients for Reliability Research. J. Chiropr. Med..

[10] Bodine R.L. (1974). Evaluation of 27 mandibular subperiosteal implant dentures after 15 to 22 years. J. Prosthet. Dent..

[11] Young L., Michel J.D., Moore D.J. (1983). A twenty-year evaluation of subperiosteal implants. J. Prosthet. Dent..

[12] Yanase R., Bodine R., Tom J., White S. (1994). The mandibular subperiosteal implant denture: A prospective survival study. J. Prosthet. Dent..

[13] Albrektsson T., Sennerby L. (1991). State of the art in oral implants. J. Clin. Periodontol..

[14] Bodine R.L., Yanase R.T., Bodine A. (1996). Forty years of experience with subperiosteal implant dentures in 41 edentulous patients. J. Prosthet. Dent..

[15] Schou S., Pallesen L., Pedersen C.S., Fibæk B., Hjørting-Hansen E. (2000). A 41-year history of a mandibular subperiosteal implant. Clin. Oral Implant. Res..

[16] Van Steenberghe D., Branemark P.-I., Quirynen M., De Mars G., Naert I. (1991). The rehabilitation of oral defects by osseointegrated implants. J. Clin. Periodontol..

[17] Esposito M., Grusovin M.G., Felice P., Karatzopoulos G., Worthington H.V., Coulthard P. (2009). The efficacy of horizontal and vertical bone augmentation procedures for dental implants—A Cochrane systematic review. Eur. J. Oral Implantol..

[18] Clark D., Barbu H., Lorean A., Mijiritsky E., Levin L. (2017). Incidental findings of implant complications on postimplantation CBCTs: A cross-sectional study. Clin. Implant. Dent. Relat. Res..

[19] Van Doorne L., Fonteyne E., Matthys C., Bronkhorst E., Meijer G., De Bruyn H. (2020). Longitudinal Oral Health-Related Quality of Life in maxillary mini dental implant overdentures after 3 years in function. Clin. Oral Implant. Res..

[20] Chiapasco M., Zaniboni M., Boisco M. (2006). Augmentation procedures for the rehabilitation of deficient edentulous ridges with oral implants. Clin. Oral Implant. Res..

[21] Gonzalez-Garcia R., Naval-Gias L., MunozGuerra M.F., Sastre-Perez J., Rodriguez-Campo F.J., Gil-Diez-Usandizaga J.L. (2005). Preprosthetic and implantological surgery in patients with severe maxillary atrophy. Med. Oral Patol. Oral Cir. Bucal..

[22] Aghaloo T.L., Misch C., Lin G.-H., Iacono V.J., Wang H.-L. (2017). Bone Augmentation of the Edentulous Maxilla for Implant Placement: A Systematic Review. Int. J. Oral Maxillofac. Implant..

[23] Simon B.I., Chiang T.F., Drew H.J. (2010). Alternative to the gold standard for alveolar ridge augmentation: Tenting screw technology. Quintessence Int..

[24] Alérico F.A., Bernardes S.R., Fontao F.G.K., Diez G.F., Alérico J.H.S., Claudino M. (2014). Prospective Tomographic Evaluation of Autogenous Bone Resorption Harvested From Mandibular Ramus in Atrophic Maxilla. J. Craniofacial Surg..

[25] Gultekin B.A., Bedeloglu E., Kose T.E., Mijiritsky E. (2016). Comparison of Bone Resorption Rates after Intraoral Block Bone and Guided Bone Regeneration Augmentation for the Reconstruction of Horizontally Deficient Maxillary Alveolar Ridges. BioMed Res. Int..

[26] Johansson B., Grepe A., Wannfors K., Hirsch J.M. (2001). A clinical study of changes in the volume of bone grafts in the atrophic maxilla. Dentomaxillofac. Radiol..

[27] Fourcade C., Lesclous P., Guiol J. (2019). Assignment of autogenous bone grafts for reconstruction of the alveolar ridge before implant placement. J. Oral Med. Oral Surg..

[28] Von Arx T., Buser D. (2006). Horizontal ridge augmentation using autogenous block grafts and the guided bone regeneration technique with collagen membranes: A clinical study with 42 patients. Clin. Oral Implant. Res..

[29] Maiorana C., Beretta M., Salina S., Santoro F. (2005). Reduction of autogenous bone graft resorption by means of bio-oss coverage: A prospective study. Int. J. Periodontics Restor. Dent..

[30] Khojasteh A., Motamedian S.R., Khojaste M. (2016). Success rate of implants placed in autogenous bone blocks versus allogenic bone blocks: A systematic literature review. Ann. Maxillofac. Surg..

[31] Al-Dajani M. (2016). Incidence, Risk Factors, and Complications of Schneiderian Membrane Perforation in Sinus Lift Surgery. Implant. Dent..

[32] Baj A., Trapella G., Lauritano D., Candotto V., Mancini G.E., Giannì A.B. (2016). An overview on bone reconstruction of atrophic maxilla: Success parameters and critical issues. J. Biol. Regul. Homeost. Agents.

[33] Bortoluzzi M.C., Cecconello R., Derech E.D., Fabris V., Manfro R. (2014). Comparative study of immediately inserted dental implants in sinus lift: 24 months of follow-up. Ann. Maxillofac. Surg..

[34] Infante A., Rodríguez C.I. (2018). Osteogenesis and aging: Lessons from mesenchymal stem cells. Stem Cell Res. Ther..

[35] Moy P.K., Aghaloo T. (2019). Risk factors in bone augmentation procedures. Periodontol. 2000.

[36] O’Connell J., Murphy C., Ikeagwuani O., Adley C., Kearns G. (2009). The fate of titanium miniplates and screws used in maxillofacial surgery: A 10 year retrospective study. Int. J. Oral Maxillofac. Surg..

[37] Cornelis M.A., Scheffler N.R., Mahy P., Siciliano S., De Clerck H.J., Tulloch J.C. (2008). Modified Miniplates for Temporary Skeletal Anchorage in Orthodontics: Placement and Removal Surgeries. J. Oral Maxillofac. Surg..

[38] Lavstedt S., Bolin A., Henrikson C.O., Carstensen J. (1986). Proximal alveolar bone loss in a longitudinal radiographic investigation I. Methods of measurement and partial recording. Acta Odontol. Scand..

[39] Bergström J., Henrikson C.O. (1970). Quantitative longitudinal study of alveolar bone tissue in man. J. Periodontal Res..

[40] Wyatt C.C. (1998). The effect of prosthodontic treatment on alveolar bone loss: A review of the literature. J. Prosthet. Dent..

[41] Kovacić I., Celebić A., Zlatarić D.K. (2010). Decreasing of residual alveolar ridge height in complete denture wearers. A five year follow up study. Coll. Antropol..

[42] Koodaryan R., Hafezeqoran A. (2016). Evaluation of Implant Collar Surfaces for Marginal Bone Loss: A Systematic Review and Meta-Analysis. BioMed Res. Int..

[43] Atwood D.A., Coy W.A. (1971). Clinical, cephalometric, and densitometric study of reduction of residual ridges. J. Prosthet. Dent..

[44] Yaffe A., Fine N., Binderman I. (1994). Regional Accelerated Phenomenon in the Mandible Following Mucoperiosteal Flap Surgery. J. Periodontol..

[45] Job S., Bhat V., Naidu E.M. (2008). In vivo evaluation of crestal bone heights following implant placement with ‘flapless’ and ‘with-flap’ techniques in sites of immediately loaded implants. Indian J. Dent. Res..

[46] Merheb J., Vercruyssen M., Coucke W., Beckers L., Teughels W., Quirynen M. (2016). The fate of buccal bone around dental implants. A 12-month postloading follow-up study. Clin. Oral Implant. Res..

[47] Nobuto T., Suwa F., Kono T., Taguchi Y., Takahashi T., Kanemura N., Terada S., Imai H. (2005). Microvascular Response in the Periosteum Following Mucoperiosteal Flap Surgery in Dogs: Angiogenesis and Bone Resorption and Formation. J. Periodontol..

[48] Merheb J., Quirynen M., Teughels W. (2014). Critical buccal bone dimensions along implants. Periodontol. 2000.

[49] Maier F.-M. (2016). Initial Crestal Bone Loss Af ter Implant Placement with Flapped or Flapless Surgery—A Prospective Cohort Study. Int. J. Oral Maxillofac. Implant..

[50] Engelbrecht W.P., Fourie Z., Damstra J., Gerrits P.O., Ren Y. (2013). The influence of the segmentation process on 3D measurements from cone beam computed tomography-derived surface models. Clin. Oral Investig..

[51] Yatabe M., Prieto J.C., Styner M., Zhu H., Ruellas A.C., Paniagua B., Budin F., Benavides E., Shoukri B., Michoud L. (2019). 3D superimposition of craniofacial imaging—The utility of multicentre collaborations. Orthod. Craniofacial Res..

